# Novel Transungual Approach Using a Cement Spacer for a Recurrent Intramedullary Glomus Tumour of the Finger: A Case Report

**DOI:** 10.7759/cureus.81764

**Published:** 2025-04-05

**Authors:** Chia Wei Ooi, Chia Hua Lim, Elaine Zi Fan Soh, Shalimar Abdullah, Jamari Sapuan

**Affiliations:** 1 Department of Orthopaedics and Traumatology, Faculty of Medicine, Universiti Kebangsaan Malaysia, Kuala Lumpur, MYS

**Keywords:** bone cement, excision, glomus tumour, intramedullary, recurrent

## Abstract

Glomus tumours are rare benign epithelial and mesenchymal neoplasms of the glomus body, which primarily occur in the subungual area of fingers, characterized by excruciating pain, point tenderness, and cold sensitivity. Glomus tumours are also reported to be extradigital in almost every organ, which makes them difficult to diagnose due to their rarity. Delayed diagnosis commonly happens due to negative imaging from plain radiograph and ultrasound imaging. Early recognition of this disease with proper diagnosis and complete surgical excision is typically effective, leading to resolution of symptoms. Despite there is a chance of recurrence even with surgical excision in some cases, probably due to incomplete excision or the presence of another undiagnosed tumour at the beginning. We present a rare case of a recurrent intramedullary glomus tumour of the finger that persisted despite multiple surgical excisions. This case was managed using a novel transungual surgical approach, incorporating a cement spacer to provide structural support for the finger pulp following the removal of the diseased distal phalanx. We also review the surgical outcomes in this challenging scenario.

## Introduction

Glomus tumours arise from glomus bodies, which are made up of glomus cells, allowing them to regulate temperature and blood pressure by controlling peripheral cutaneous blood flow, mainly found in palms, digits, and soles. It accounts for 4.5% of upper limb soft tissue tumours, with the most common site around 50% in the subungal region [1]. A common clinical presentation involves triad symptoms of pinpoint tenderness, pain, and cold hypersensitivity, which leads to a clinical diagnosis in most cases. Studies showed that symptoms typically persisted for an average of seven to 11 years, with patients having an average of two to three consultations before receiving a diagnosis [2]. Magnetic resonance imaging (MRI) remains as gold standard investigation to assist in accurately identifying the location, margin, and depth of glomus tumors before operation [3]. Fazwi et al. stated in their study that the average recurrence rate post-surgical excision was 13% [2]. The reason for its recurrence within a year is mainly due to inadequate excision, while delayed recurrences after a year are presumed possible from concurrent satellite lesions that were overlooked during the initial surgery, leading to the formation of a new tumour near the excision site [4].

## Case presentation

A 37‐year‐old woman initially presented in 2020 with a two‐year history of left index finger pain, marked by hypersensitivity, cold intolerance at the fingertip, and associated swelling. She denied any history of trauma. An initial ultrasound was unremarkable, and a diagnosis of a glomus tumour of the left index finger was established. She underwent surgical excision in February 2020. Intraoperatively, a whitish tumour measuring 4 × 2 mm was identified over the distal phalanx with a cortical breach on the volar aspect. Histopathology confirmed the diagnosis of a glomus tumour.

Approximately 18 months later, she experienced a recurrence of pain in the same finger. Clinical examination revealed tenderness at the distal phalanx with a positive Love’s test. Plain radiographs and a repeat ultrasound did not demonstrate any abnormalities. However, an MRI revealed a recurrent subungual glomus tumour at the distal phalanx, characterized by T1-weighted hypointensity and T2-weighted hyperintensity.

In 2023, a second excision was performed via a transungual approach under ring block anaesthesia. Intraoperative findings included a 2 × 2 mm glomus tumour beneath the nail bed, indenting the bone of the distal phalanx, a glomus tumour with appearance of gelatinous such as tissue, was curretaged. Tissue samples obtained from the nail bed confirmed the presence of a glomus tumour, while the sample from the intramedullary space suggested incomplete excision. The patient was informed about the incomplete resection and the potential for recurrence, and conservative management was subsequently chosen.

**Figure 1:**
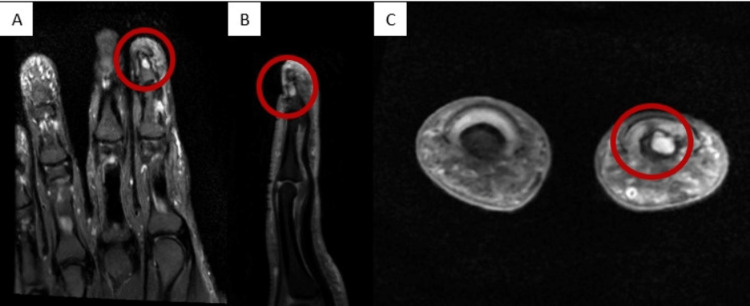
Magnetic resonance imaging: coronal view (A), sagittal view (B), and axial view (C). A well-defined lesion was seen at the distal phalanx of the left index finger within the intramedullary region, measuring 2.7 x 2.1 x 3.2 mm, demonstrating hyperintense signal in T2W (circled with a red marker).

Six months later, in early 2024, she returned with persistent, excruciating pain in the left index finger, accompanied by hypersensitivity and nocturnal pain. Examination revealed a vertical ridge across the entire nail plate with pinpoint tenderness. A repeat MRI demonstrated a well-defined lesion within the intramedullary portion of the distal phalanx, measuring 2.7 × 2.1 × 3.2 mm, with T1-weighted hypointensity and T2-weighted hyperintensity (Figures 1A-1C).

Treatment

Given the multiple recurrences of the glomus tumour at the same site - with the unusual involvement of the intramedullary region - the patient was advised to undergo partial resection of the involved distal phalanx with bone cement insertion via a transungual approach.

Preoperative Preparation

The left index finger was anaesthetized using a ring block with 2% lignocaine, and a digital tourniquet was applied.

Surgical Procedure

A dorsal incision was made along the proximal nail fold, and the nail plate was completely removed to expose the underlying nail bed. 

**Figure 2:**
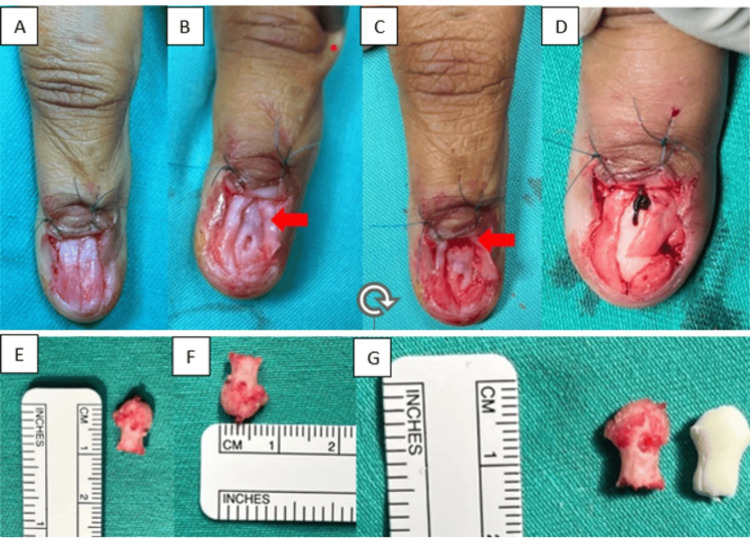
Intraoperative images of the excision of the glomus tumour using the transungal approach. (A) Removal of the nail plate with longitudinal incision in the midline of the nail bed. (B) Bluish discoloration was seen over the dorsal cortex of the distal phalanx with defect from the previous surgical excision after dissecting the nail bed. (C) The diseased distal phalanx was excised distal to the flexor and extensor tendon attachment. (D) Complete excision of the diseased distal phalanx. (E-F) Diseased distal phalanx of the left index finger excised measuring 0.8 x 0.9 cm. (G) Bone cement was molded into shape of the tip of the distal phalanx and fill up the defect over the finger pulp.

A longitudinal midline incision was then performed in the nail bed (Figure 2A). Bluish discoloration was observed on the dorsal cortex of the distal phalanx, corresponding to the defect from the previous excision (Figure 2B). The diseased segment of the distal phalanx was excised completely, distal to the flexor and extensor tendon attachments (Figure 2C). The remaining distal phalanx stump was curetted and irrigated with hydrogen peroxide and alcohol to ensure clear tumour margins (Figure 2D). The excised specimen measured 0.8 × 0.9 cm (Figures 2E-2F).

Reconstruction

**Figure 3:**
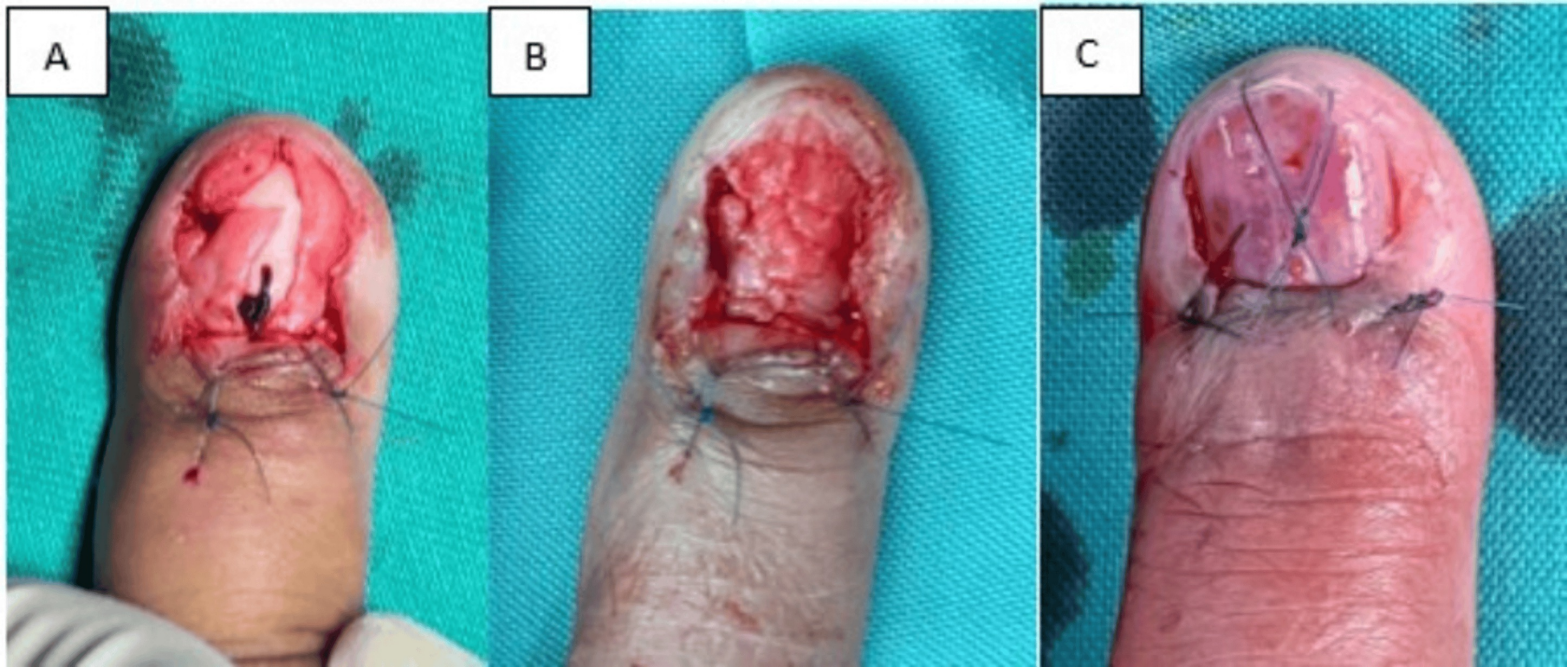
(A) Placement of the bone cement to fill the void post excision of the distal phalanx. (B) Repair of the nail bed with Monosyn 6/0. (C) Nail plate anchored with a figure of 8 suture with Dafilon 4/0.

To fill the bone defect at the fingertip, bone cement was molded to replicate the contour of the distal phalanx (Figure 2G) and inserted into the void (Figure 3A). The nail bed was approximated using Monosyn 6/0 sutures (Figure 3B), and the nail plate was secured with a figure-of-eight suture using Dafilon 4/0 (Figure 3C).

**Figure 4:**
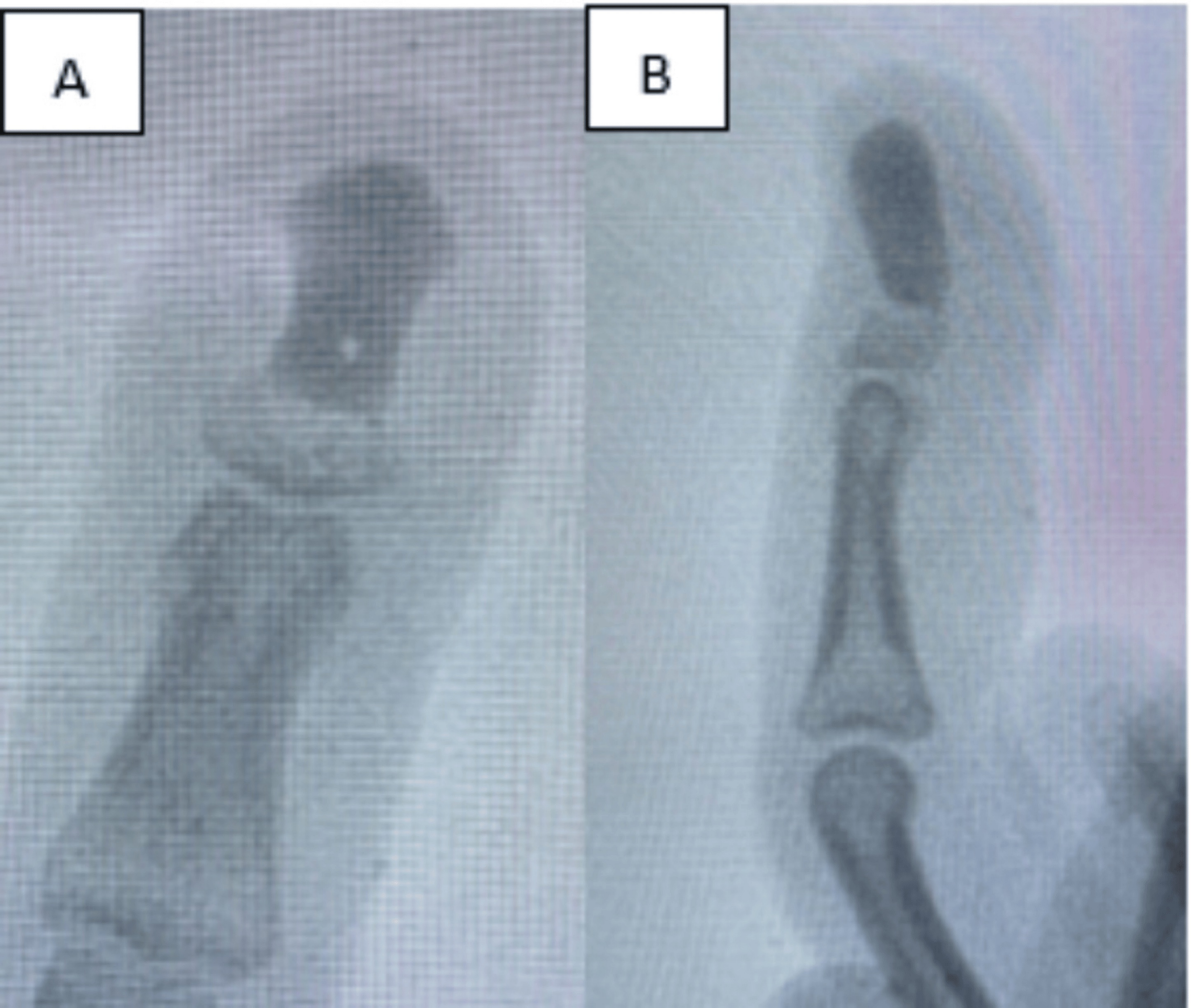
(A and B): Intensifier image of the left index finger with the proper placement of the bone cement over the tip of the distal phalanx supporting the pulp.

Postoperative imaging confirmed the proper placement of the bone cement over the distal phalanx, effectively supporting the pulp (Figure 4A and Figure 4B).

Postoperative Outcome

**Figure 5:**
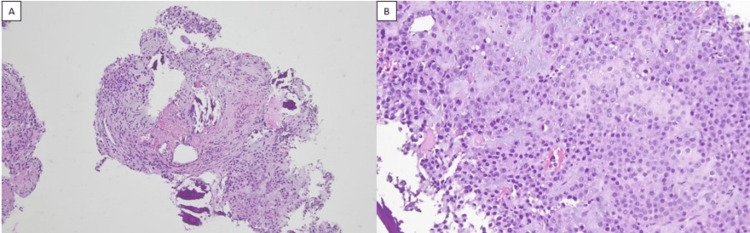
Histopathology slide of the resected distal phalanx. (A) 2x magnification, (B) 40x magnification showing fragments of the tumor tissue composed of clusters of neoplastic cells exhibiting a uniform, monomorphic round nuclear contour with focal myxoid stroma consistent with a glomus tumour.

The patient recovered well with complete resolution of fingertip pain and was discharged with follow-up arrangements in the clinic. Histopathological analysis of the resected distal phalanx confirmed a glomus tumour (Figure 5A and Figure 5B).

Follow-up

**Figure 6:**
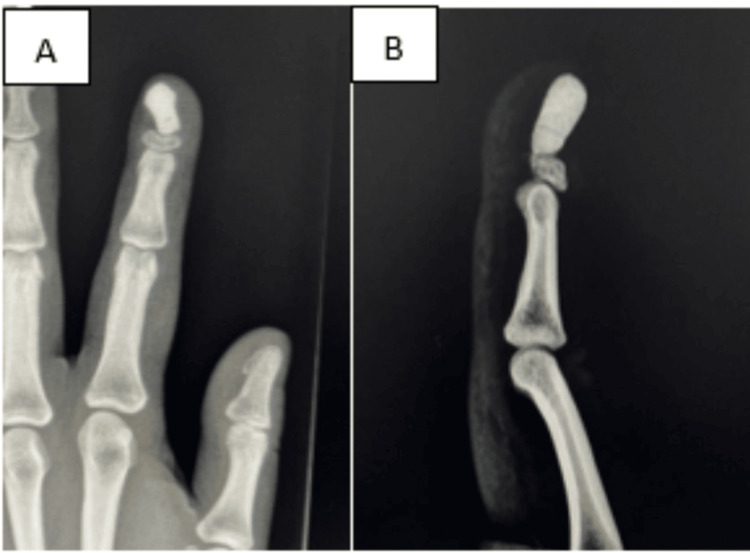
(A and B): Plain radiograph of left index finger showing malalignment of the bone cement over the distal phalanx impinging over the dorsal surface of the fingertip.

At the six‐week postoperative follow-up, the patient reported recurrent hypersensitivity and pain at the fingertip, which appeared to be due to the bone cement abutting the tip. The wound was otherwise well healed with no signs of infection. A plain radiograph of the left index finger revealed malalignment of the bone cement over the distal phalanx, impinging on the dorsal surface of the fingertip (Figures 6A-6B).

After discussing the situation with the patient - and considering the multiple previous procedures and persistent symptoms - it was agreed that disarticulation of the distal phalanx would provide the best chance for symptom relief. Consequently, the distal phalanx was disarticulated at the distal interphalangeal joint via a fish-mouth incision.

Histopathological analysis of the amputated specimen, which included the remaining base of the distal phalanx and nail bed, revealed a foreign body reaction and chronic inflammation, with no residual glomus tumour identified.

The patient was followed up once one month after the operation in our hand clinic. During review, the patient was well and symptom-free, and she was satisfied with the outcome and discharged from the hand clinic without further follow-up. Otherwise, the patient also did not return to us with new symptoms.

## Discussion

**Figure 7:**
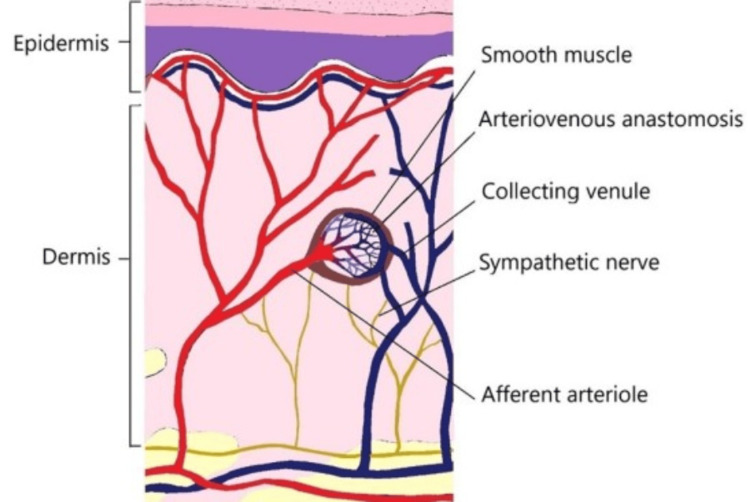
A schematic diagram of the glomus body in the dermis layer of the skin. Source: Ref [5]

Glomus tumours arise from the glomus body, a neuromyoarterial structure involved in thermoregulation, and are most commonly found in the subungual areas of digits (Figure 7) [5]. These tumours are commonly encountered by hand surgeons due to their frequent occurrence in the fingers, with the highest incidence reported at 61% in Lee et al.'s study [6]. Extradigital glomus tumours represent 47% of cases, as described by Fazwi et al. [2]. The condition predominantly affects women between 20 and 40 years of age [7].

Although glomus tumours are generally considered benign, rare cases of malignant transformation have been documented [8]. Clinically, glomus tumours classically present with the Carroll triad, which includes paroxysmal pain, cold sensitivity, and localized tenderness over the affected digit. A small pink or bluish nodule may also be visible in the subungual region. Several diagnostic tests can help confirm the diagnosis, including Love’s test, Hildreth’s test, transillumination, and cold tests, each with variable sensitivity and specificity [9]. Love's test is a clinical diagnostic tool for glomus tumors, where pinpoint pressure over the suspected area will cause excruciating pain, confirming the affected area. A positive Hildreth' test means that inflation of the tourniquet up to 250 mmHg in the arm will abolish tenderness over the palpated area and the recurrence of pain after releasing pressure from the tourniquet. On average, the time from symptom onset to a definitive diagnosis is approximately 44 months [6]. Delays in diagnosis are primarily due to a lack of awareness and experience with this rare condition. Therefore, it is crucial for clinicians to maintain a high index of suspicion when encountering such cases and base their diagnosis on a thorough history and physical examination. Differential diagnoses to consider include neuroma, pyogenic granuloma, osteomyelitis, lipoma, cyst, paronychia, and haemangioma [2].

Glomus tumours are soft tissue lesions and typically do not involve the bony cortex. However, intraosseous involvement was first reported by Iglesias in 1939 [10]. Long-standing pressure from the tumour on the underlying bone can lead to scalloping and concavity, particularly on the cortex of the distal phalanx [1]. In this case, MRI revealed that the glomus tumour involved the intramedullary space of the distal phalanx, which was not detected by plain radiographs or ultrasound. Doppler ultrasonography has been shown to be a useful diagnostic tool due to the hypervascular nature of glomus tumours; however, it is operator dependent.

On MRI, glomus tumours typically appear as well-defined, dark masses on T1-weighted images, hyperintense on T2-weighted images, and brightly enhancing after gadolinium contrast administration. These features allow differentiation from other cysts and tumours of the hand. Therefore, MRI plays a critical role in preoperative planning, enabling precise identification of tumour location, size, and the presence of multifocal lesions, thus reducing the risk of recurrence [10].

Complete surgical excision is considered curative and provides immediate relief from symptoms, including psychological distress [11]. Fazwi et al. reported a recurrence rate of 4%-15% after excision [2]. Early recurrence within a year is often due to incomplete removal or the presence of undiagnosed satellite lesions that were not excised during the initial procedure. In our case, the recurrence likely resulted from incomplete excision of the tumour during the first operation, as the surgery was performed prior to MRI, which would have allowed for better delineation of the lesion’s depth and location. A subsequent recurrence was attributed to inadequate removal of the tumour from the intramedullary region, which is a rare manifestation of glomus tumour with intraosseous involvement, as evidenced by histopathology of the tissue from the distal phalanx intramedullary space showing incomplete excision. Awaiting further symptom recurrence, we ultimately decided to proceed with complete extirpation by removing the distal portion of the distal phalanx invaded by the glomus tumour to prevent additional recurrences.

Several surgical approaches for glomus tumour excision have been reported in the literature, including the transungual, Keyser-Littler, lateral subperiosteal, modified periungual, and nail bed margin approaches [12]. Nam et al. suggested a nail-sparing and sub-nail bed approach for improved aesthetic outcomes, reduced postoperative pain, and higher patient satisfaction [13]. The transungual approach is widely recognized for providing optimal exposure and easy access to the tumour; however, it carries the risk of injury to the nail bed and germinal matrix, potentially resulting in nail deformities. To minimize this risk, the lateral subperiosteal and Keyser-Littler approaches were developed to preserve the nail bed. The Keyser-Littler technique is a lateral approach through high mid-lateral entry just beneath the paronychial fold whereby the distal phalangeal ligament is retracted and the nail matrix is lifted over the ligament and the dorsal cortex of the distal phalanx. In our case, we utilized the transungual approach with an H-incision over the proximal nail fold. The nail plate was removed, and a longitudinal midline incision was made in the nail bed in order to fully visualize the distal phalanx due to the multiple recurrences. After excising the distal portion of the distal phalanx, the remaining stump was curetted, and bone cement was used to fill the resultant cavity, providing structural support to the nail bed and preventing collapse. However, there is a micro-movement between the bone cement spacer and the remaining proximal portion of the distal phalanx, which was not anchored, just held up by the repair of the nail bed, which had ultimately caused a delayed complication of toggling of the bone cement within the cavity.

Studies have shown that using bone cement post-curettage reduces recurrence risk in giant cell tumors compared to bone grafting [14]. Moreover, the exothermic reaction during bone cement polymerization helps destroy any residual tumour cells and avoids donor site morbidity. To our knowledge, this is the first reported case using bone cement in glomus tumour excision for defect management and to provide stability for early rehabilitation. Although the immediate postoperative outcome was promising, with reduced pain and improved patient satisfaction, a delayed complication arose. The remaining bony stump eventually resorbed, causing the bone cement to toggle and impinge on the nail bed, leading to pain and hypersensitivity. Ultimately, this necessitated disarticulation.

The key lessons from this case underscore the importance of meticulous and complete surgical excision for definitive symptom relief and cure, as well as the critical role of preoperative MRI in confirming the exact location and depth of the tumour - especially in rare cases with intramedullary involvement - to guide the surgical approach.

## Conclusions

Intramedullary, glomus tumours of the finger, although rare, should be considered in patients with unexplained nail pain. Preoperative MRI play a crucial role in helping delineate the proper margin of the tumour extent for complete excision. The transungual approach provides excellent visualization for tumor excision, and meticulous, complete resection is essential to minimize the risk of recurrence.
